# Utility of repeating bone marrow biopsy for confirmation of complete response in multiple myeloma

**DOI:** 10.1038/s41408-020-00363-6

**Published:** 2020-10-02

**Authors:** Marcella A. Tschautscher, Dragan Jevremovic, Francis K. Buadi, Martha Q. Lacy, Morie A. Gertz, Angela Dispenzieri, Prashant Kapoor, David Dingli, Lisa Hwa, Amie Fonder, Miriam Hobbs, Suzanne Hayman, John Lust, Stephen J. Russell, Nelson Leung, Ronald S. Go, Yi Lin, Wilson Gonsalves, Taxiarchis Kourelis, Rahma Warsame, Robert A. Kyle, S. Vincent Rajkumar, Shaji Kumar

**Affiliations:** 1 Division of Hematology, Mayo Clinic, Rochester, MN USA; 2 Division of Hematopathology, Mayo Clinic, Rochester, MN USA; 3 Division of Hematology and Molecular Medicine, Mayo Clinic, Rochester, MN USA; 4 Division of Nephrology and Hypertension, Department of Internal Medicine, Mayo Clinic, Rochester, MN USA

**Keywords:** Myeloma, Myeloma

## Introduction

Achievement of complete response (CR) in multiple myeloma (MM) has been defined by the International Myeloma Working Group (IMWG) as concurrent demonstration of disappearance of monoclonal protein in the serum and urine with negative immunofixation (IFE), in addition to a bone marrow biopsy demonstrating < 5% bone marrow plasma cells (BMPC)^1,2^. With advancements in myeloma-directed therapies and increasing CR rates, it is imperative that accurate and uniform application of CR criteria be utilized, especially with increased incorporation of minimal residual disease assessment^3,4^. While obtaining serum and urine samples for monoclonal protein analyses are simple, bone marrow biopsies are associated with pain, are inconvenient, and are burdensome to patients.

We are occasionally confronted with the scenario of the bone marrow fulfilling the criterion for CR (<5% PCs) with simultaneous positive serum or urine immunofixation. In these circumstances, a repeat bone marrow biopsy would be required at the time of serum and urine monoclonal protein disappearance, subjecting patients to another painful and uncomfortable procedure that is unlikely to yield different results or influence clinical decision making. We, therefore, investigated the utility of repeating a BM biopsy at the time of serum and urine IFE negativity in myeloma patients undergoing autologous stem cell transplant (ASCT) with prior marrow demonstrating <5% PCs.

## Patients and methods

A retrospective cohort study was conducted on all myeloma patients seen at Mayo Clinic who underwent an autologous stem cell transplant between 1998 and 2016. Patients included in the analysis had to have a pre-transplant BM biopsy demonstrating <5% PCs with concomitant serum and urine IFE studies and post-transplant bone marrow biopsy, serum, and urine IFE studies available for analysis. Frequency and degree of BMPC clonality were recorded in the pre-transplant BM biopsy. Serum and urine immunofixation status, collected within 30 days of the pre-transplant bone marrow biopsy was recorded as positive or negative. Among patients who had a positive pre-transplant serum or urine IFE, post-transplant data (day 100 after SCT) including BMPC percentage, serum IFE, and urine IFE were recorded, with all IFE collections occurring within 30 days from the bone marrow biopsy. We then examined the proportion of patients in whom the serum and urine IFE became negative and its association with BMPC percentages. All statistical analyses, storage of data, and image generation were performed using the JMP 14.1.0 statistical package (SAS Institute Inc., Cary, NC). Approval for this study was obtained from the Mayo Clinic IRB and informed consent was obtained from all patients for review of their medical records.

## Results

The median time from the pre-transplant bone marrow biopsy to date of transplant was 0.53 months (0.2–5 months). We identified 277 patients in our database with pre-transplant IFE positivity and pre-transplant BMPC <5%with post-transplant data available for analysis. Among these patients, 179 (64.6%) patients had detectable clonal plasma cells in the pre-transplant BM biopsy, with a median clonal PC percentage of 0.55% (0.1%-29%). Following the transplant, 116/277 (42%) patients were found to meet IFE criteria for CR (i.e. IFE negative in both serum and urine) while 161 (58%) patients remained IFE positive post-transplant in either the serum or urine (VGPR).

**Fig. 1:**
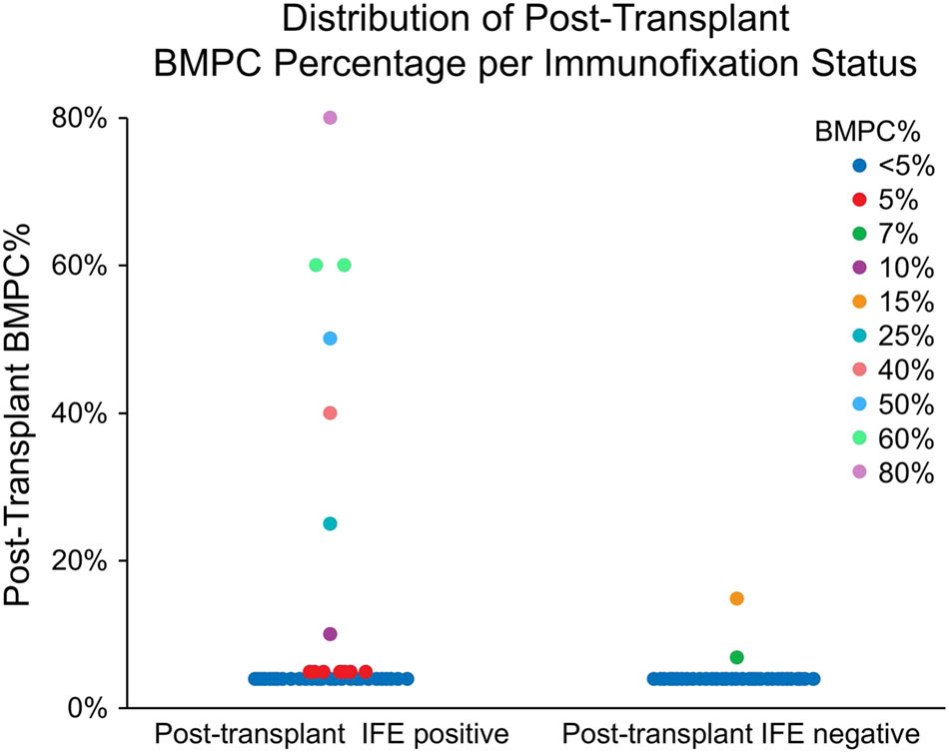
Post-transplant bone marrow biopsy plasma cell percentage with corresponding post-transplant immunofixation status among patients proceeding to ASCT with bone marrow plasma cells <5%, but IFE positivity. All patients represented in scatter plot met inclusion parameters including pre-transplant BMPC < 5% and pre-transplant serum or urine IFE positivity (*n* = 277). Among patients with post-transplant IFE positivity, 14 had BMPC > 5%. Among those with post-transplant IFE negativity, 2 had BMPC > 5%.

A repeat marrow examination was done at a median of 3.3 months (1.7–4.9 months) from transplant. Among the patients with negative post-transplant serum and urine IFE (*n* = 116), 98% had unchanged BMPC percentage post-transplant (BMPC < 5%) and the remaining 2 patients demonstrated BMPC compositions of 15% and 7%, both of which had 6% clonal plasma cells. In contrast, among the cohort of patients who were IFE positive post-transplant, 14 (8.7%) demonstrated an increase in PC composition in their post-transplant bone marrow biopsies, with a median BMPC cellularity of 7.5% (5–80%) among which 12 had detectable clonal plasma cells with a median of 5.6% (0.2–41.2%), (Fig. 1). The remaining 94% of patients had unchanged BMPC composition (Fig. 1).

## Discussion

Based on current IMWG criteria for confirming CR in myeloma, a patient must demonstrate serum and urine IFE negativity with concomitant bone marrow findings of <5% plasma cells^1^. While the timing in concordance of these parameters allows for only one bone marrow biopsy, we are occasionally confronted with conflicting results, specifically with a bone marrow biopsy fulfilling criteria for CR with contradictory peripheral studies. In these cases, IMWG guidelines would suggest repeating a bone marrow biopsy at the time when both serum and urine IFE become negative, thus subjecting the patient to another distressing and painful procedure^1^.

The results of our study demonstrate that in 98% of patients whose bone marrow biopsy met criteria for CR before immunofixation studies (negative urine and serum IFE), there was no difference in the BMPC percentage with a repeat biopsy after ASCT. One explanation for this phenomenon is the difference in the monoclonal protein clearance kinetics and BMPC composition, with a delay in disappearance of M protein relative to marrow disease clearance^5^. Among the remaining patients with available post-transplant serum and urine IFE not otherwise meeting criteria for CR, 14 (8.7%) demonstrated an increase in the composition of BMPC from <5% pre-transplant. This finding could be explained by myeloma’s patchy marrow involvement which is reflected in the initial, falsely reassuring bone marrow biopsy with true disease activity captured upon subsequent marrow sampling^6^. This rationale is supported by the presence of clonal plasma cells in the post-transplant BM biopsy in this cohort, detected in 12 of 14 patients. Similarly, the observed inconsistency could also be due to an aggressive disease phenotype with progression occurring near day 100, however in these settings serum and/or urine IFE were in accordance with BM findings. The limitation in relying solely on the bone marrow biopsy is further supported with the finding of positive immunofixation either in the serum, urine, or both in these clinical settings. This reinforces the need and utility of all three parameters to accurately confirm a state of CR, however not necessarily simultaneously.

One limitation of our study is this analysis only evaluated IMWG criteria for CR and not stringent complete response (sCR), which requires serum free light chain assessment and the absence of clonal plasma cells via immunohistochemistry. Secondly, we do not provide details regarding the status of soft tissue plasmacytomas via imaging to correlate with our findings. Finally, while 64% of patients had detectable clonal plasma cells in the pre-transplant biopsy, in general the proportion of clonal PCs in these samples were found at low prevalence (median 0.55%), the relevance of which has been shown to correlate with improvement in clinical outcomes, especially if present at <5%^7^.

Our findings suggest that in the setting where pre-myeloma directed therapy serum or urine IFE lag in fulfilling CR criteria with BMPC <5%, repeating a bone marrow biopsy after treatment is unnecessary and unlikely to provide any additional information and influence approach to care outside of the clinical trial setting. This would save many myeloma patients from unneeded biopsies if added to the current IMWG complete response criteria.
